# Supplementary material for: Characterization of Human Senescent Cell Biomarkers for Clinical Trials
Source: Aging Cell. 2025 Jan 17;24(5):e14489. doi: 10.1111/acel.14489 (PMC12073900; doi:10.1111/acel.14489)
Supplement: Supplementary file 1 — Data S1. [file ACEL-24-e14489-s001.pdf]

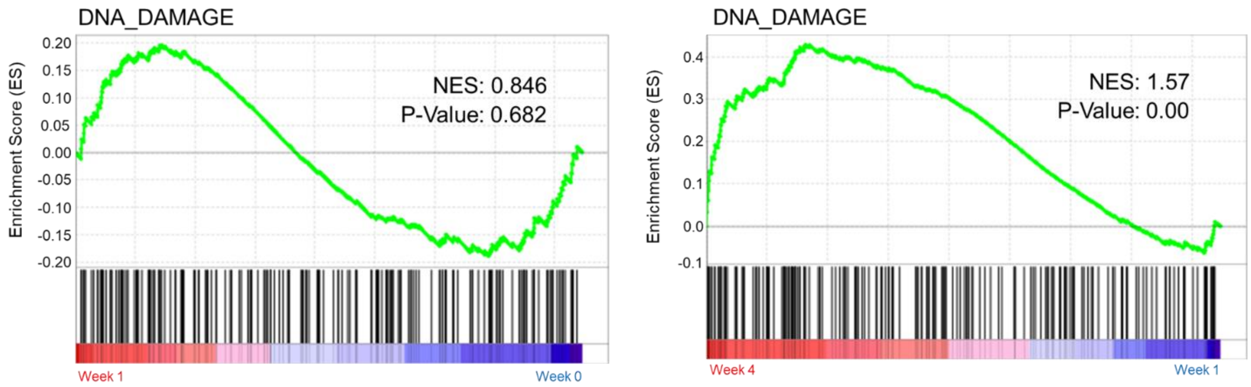

Figure S1. GSEA analysis using a DNA damage gene set. The Kyng_DNA_Damage_Up gene set (MSigDB) was used to compare the DNA damage response in the IMR90 cells following exposure to etoposide. Left panel shows the comparison of week 1 to week 0 and the right panel shows the comparison of week 4 to week 1. n=5 week 0, n=6 weeks 1 and 4.

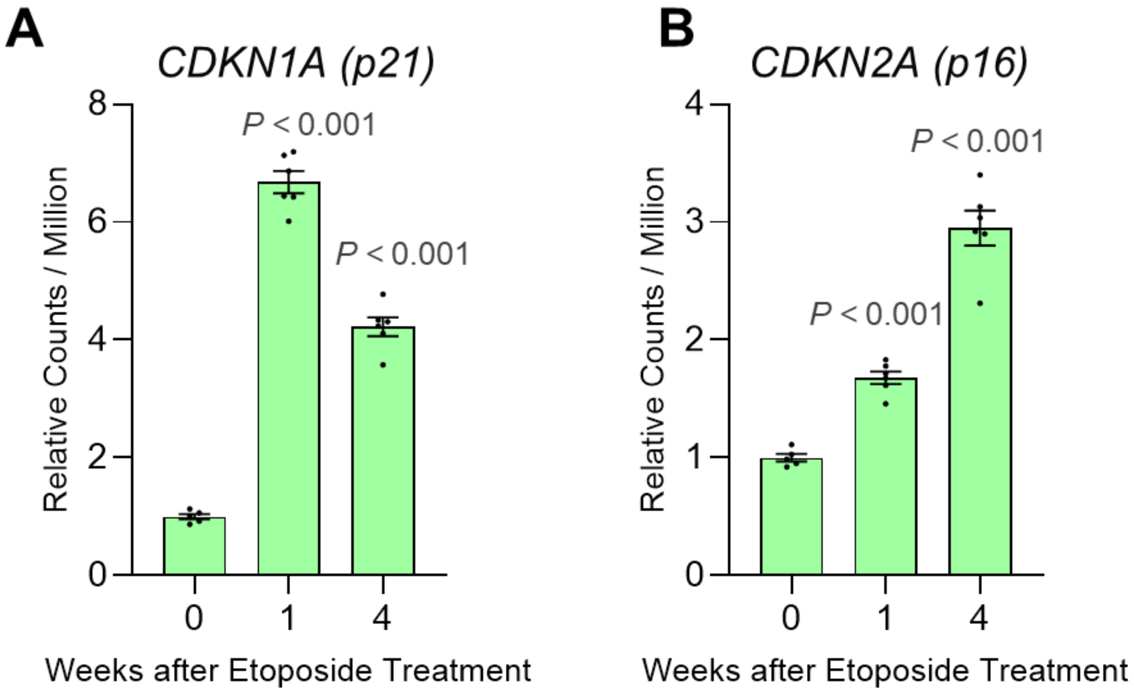

Figure S2. Changes in *p21*^{Cip1} and *p16* mRNA levels in IMR90 cells. Cells were exposed to etoposide to induce DNA damage and expression of (A) *p21*^{Cip1} mRNA levels and (B) *p16* mRNA levels were assessed using RNAseq at baseline (week 0) and weeks 1 and 4. n=5 week 0, n=6 weeks 1 and 4. Data are mean ± SEM; *P*-values based on 2-sided t-test.

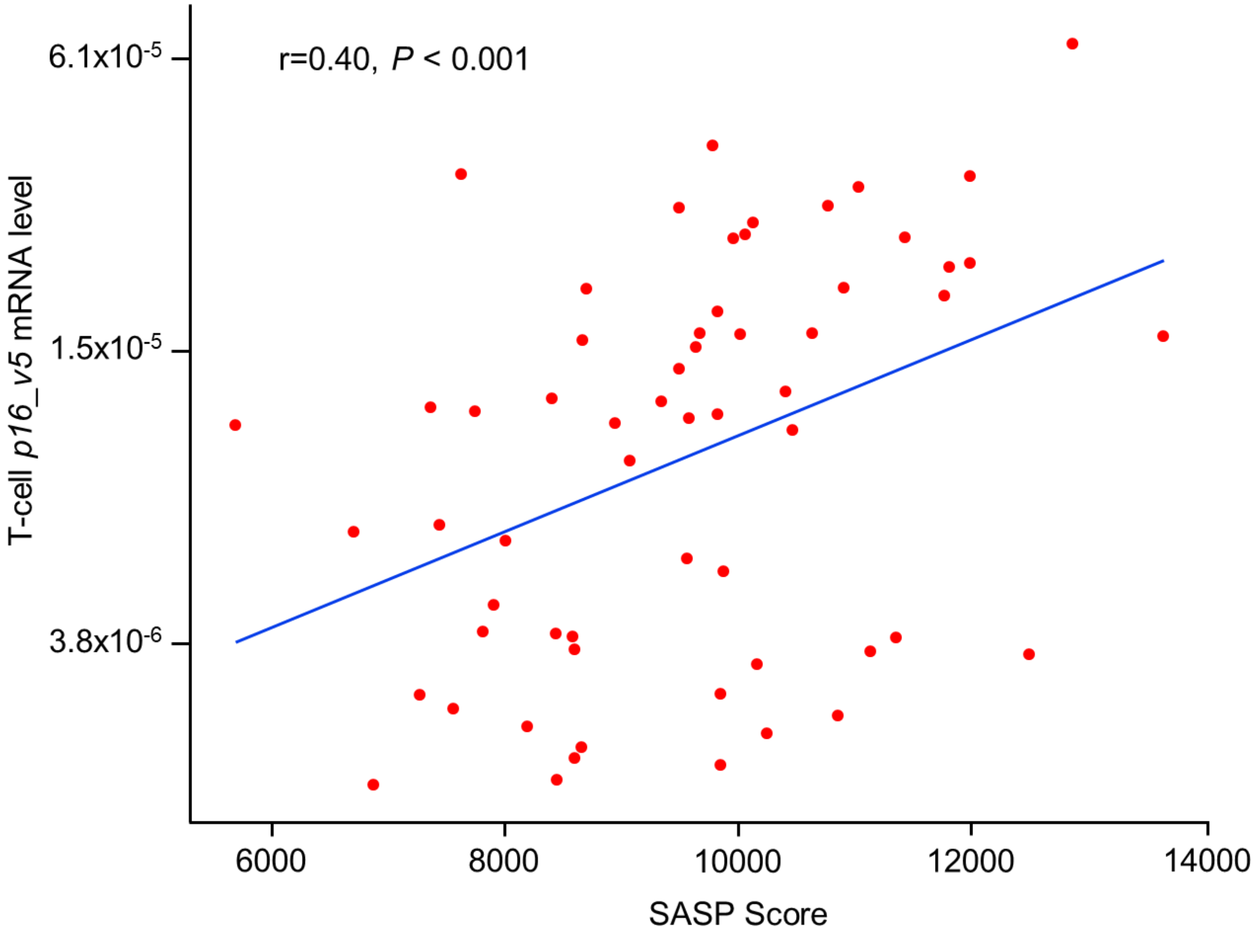

Figure S3. Correlation between the SASP score and T-cell *p16_variant 5* mRNA levels. N=60, Spearman correlations.

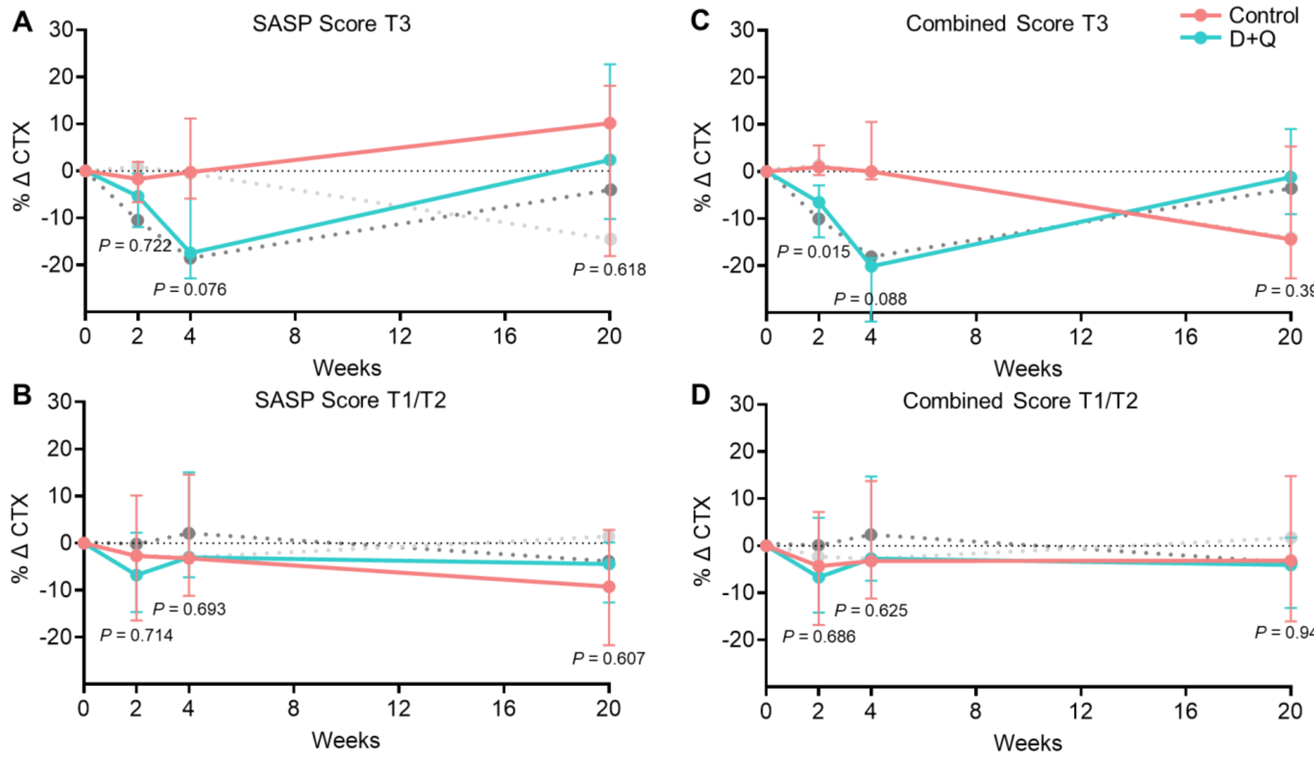

Figure S4. Time course of changes in the bone resorption marker, serum CTx, based on the SASP score tertiles. (A) Participants in the highest tertile for the SASP score (T3). (B) participants in the lower two tertiles for the SASP score (T1/T2), n=21 T3 and 39 T1/T2 at 2 weeks; n=20 T3 and 39 T1/T2 at 4 weeks; n=18 T3 and 38 T1/T2 at 20 weeks. (C) participants in the T3 group for both the SASP score and T-cell *p16_variant 5* mRNA levels, and (D) participants in the T1/T2 groups for either the SASP score and T-cell *p16_variant 5* mRNA levels. n=13 T3 and 47 T1/T2 at 2 weeks; n=12 T3 and 47 T1/T2 at 4 weeks; n=10 T3 and 46 T1/T2 at 20 weeks. In each panel, shaded lines show the corresponding changes based on using the T-cell *p16_variant 5* mRNA for stratification, as per our previous analysis in Farr et al. (Farr et al., 2024) (lighter shade is control, darker shade is D+Q). Data are shown as Median (IQR); *P*-values based on two-sided Wilcoxon rank-sum tests.

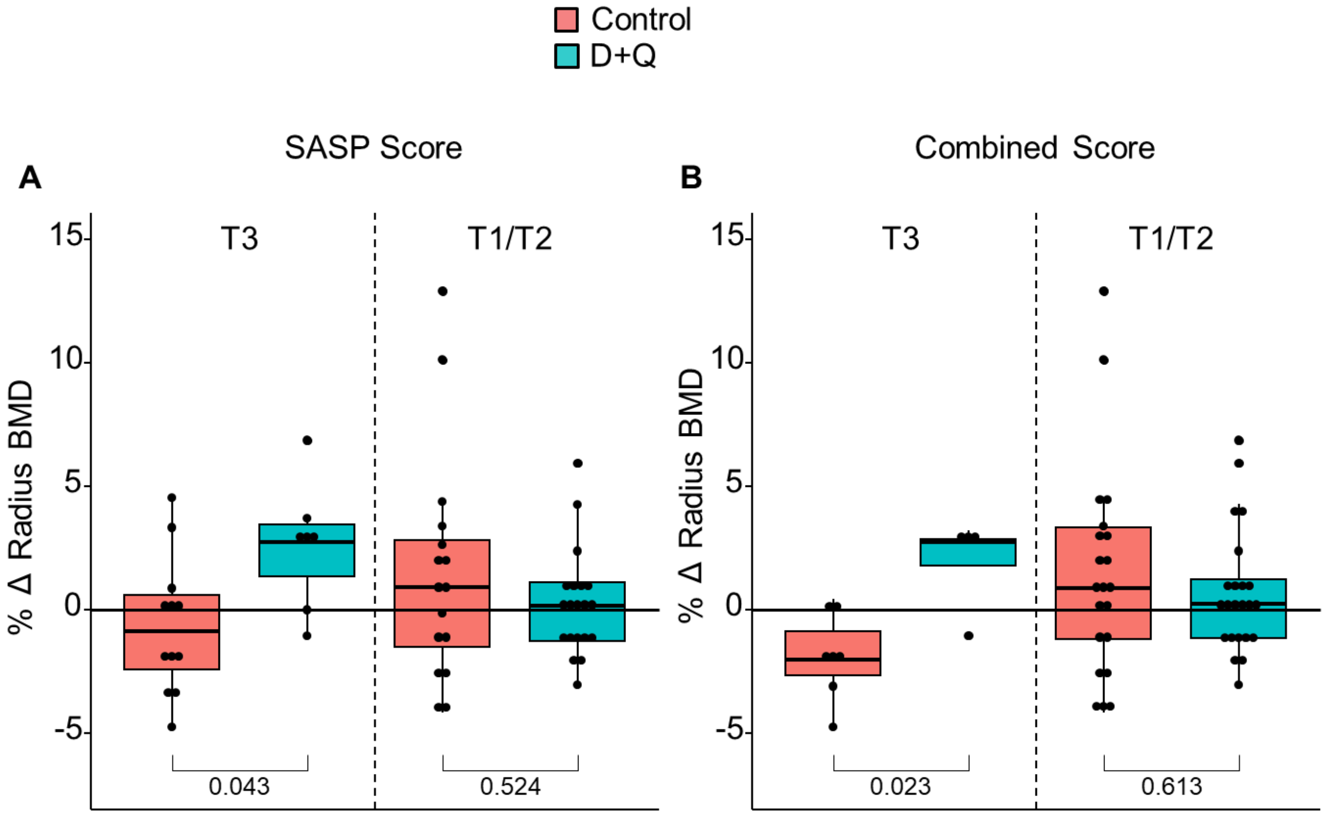

Figure S5. Changes in radius BMD at 20 weeks based on the SASP score tertiles. (A) Participants in the highest tertile for the SASP score (T3) versus participants in the lower two tertiles for the SASP score (T1/T2), n=19 T3 and 38 T1/T2. (B) participants in the T3 group for both the SASP score and T-cell *p16_variant 5* mRNA levels versus participants in the T1/T2 groups for either the SASP score and T-cell *p16_variant 5* mRNA levels, n=11 T3 and 44 T1/T2. Data are shown as Median (IQR); *P*-values based on two-sided Wilcoxon rank-sum tests.

Table S1. qPCR primer sequences used in the study.

Gene	Description	Forward Primer Sequence	Reverse Primer Sequence
ACTB	Housekeeping	CCCAGCCATGTACGTTGCTAT	TCACCGGAGTCCATCACGAT
CDKN2A_variant 5	*p16_variant 5*	CAGAAATGATCGGAAACCATT	CTACGCATGCCTGCTTCTAC
CDKN2A_variant 1+5	*p16_variant 1+5*	CCAACGCACCGAATAGTTACG	GCGCTGCCCATCATCATG
